# Molybdate uptake interplay with ROS tolerance modulates bacterial pathogenesis

**DOI:** 10.1126/sciadv.adq9686

**Published:** 2025-01-15

**Authors:** Min Jiao, Wenbo He, Zhenlin Ouyang, Qinyue Yu, Jiaxin Zhang, Qian Qin, Ruochen Wang, Xiaolong Guo, Ruihan Liu, Xiaoyu He, Peter M. Hwang, Fang Zheng, Yurong Wen

**Affiliations:** ^1^ Center for Microbiome Research of Med-X Institute, Shaanxi Provincial Key Laboratory of Sepsis in Critical Care Medicine, The First Affiliated Hospital, Xi’an Jiaotong University, Xi’an 710061, China.; ^2^ The Key Laboratory of Environment and Genes Related to Disease of Ministry of Education Health Science Center, Xi’an Jiaotong University, Xi’an 710061, China.; ^3^ Departments of Medicine and Biochemistry, Faculty of Medicine & Dentistry, University of Alberta, Edmonton, Alberta T6G 2R3, Canada.

## Abstract

The rare metal element molybdenum functions as a cofactor in molybdoenzymes that are essential to life in almost all living things. Molybdate can be captured by the periplasmic substrate-binding protein ModA of ModABC transport system in bacteria. We demonstrate that ModA plays crucial roles in growth, multiple metabolic pathways, and ROS tolerance in *Acinetobacter baumannii*. Crystal structures of molybdate-coordinated *A. baumannii* ModA show a noncanonical disulfide bond with a conformational change between reduced and oxidized states. Disulfide bond formation reduced binding affinity to molybdate by two orders of magnitude and contributes to its substrate preference. ModA-mediated molybdate binding was important for *A. baumannii* infection in a murine pneumonia model. Together, our study sheds light on the structural and functional diversity of molybdate uptake and highlights a potential target for antibacterial development.

## Teaser

Molybdate uptake in *A. baumannii* interplay with ROS through ModA uses a noncanonical disulfide bond and modulates pathogenesis.

## INTRODUCTION

For pathogenic bacteria, success in colonization and proliferation in the host depends on obtaining nutrients like transition metal elements, of which molybdenum ranks third following iron and copper (*1*). Molybdenum is bioavailable as molybdate (MoO_4_^2−^) and is incorporated into the molybdenum cofactor (MoCo), which can be incorporated into various molybdoenzymes, redox-active enzymes involved in nitrogen, sulfur, and carbon metabolism (*2*–*4*). Substrate availability, oxygen availability, and the cellular concentrations of molybdenum and iron affect the regulation of molybdoenzyme synthesis in *Escherichia coli* (*5*). Molybdoenzymes are evolutionarily ancient and can be traced back to the last universal common ancestor (*6*). They exist widely in most life forms and are especially abundant in bacteria, with over 50 distinct types of enzymes identified (*4*).

To uptake molybdate, bacteria are equipped with several permeases and importer systems, among which ModABC is a high-affinity transporter (*7*, *8*). The *modABC* operon encodes ModA, ModB, ModC, and a regulator ModE. ModA is a soluble substrate binding protein, responsible for the acquisition of MoO_4_^2−^, the first step in transport. ModB is a transmembrane protein that receives MoO_4_^2−^ from ModA and transports it across the cell membrane with the support of adenosine 5′-triphosphate (ATP) hydrolysis by ModC, which has a nucleotide binding domain (*9*–*11*). As group six metal oxyanions, molybdate, and tungstate have similar sizes, while the molecular mechanisms behind the different kinetic values of ModA are needed to be explored, although there has been structural bias with larger substrate binding pocket of choice for molybdate instead of phosphate radical or sulfate radical (*12*, *13*), the evolutionary theory of bacteria or archaea of habitat from ocean to land, changing obtainability of two substrates, and dependence on oxygen from anaerobe to aerobe (*14*).

Molybdate import system failure can lower its availability for incorporation into MoCo, leading to a reduction or even loss of molybdoenzyme activities and decreased environmental fitness (*15*). ModABC is essential for host infection, and it is rendered defective in molybdate assimilation by *modA* mutation (*16*). ModA in *Acinetobacter baumannii* has been identified as a periplasmic protein (*17*), whereas in *Pseudomonas aeruginosa*, ModA was shown to be secreted by H2-T6SS and to interact with the outer membrane protein IcmP to deliver molybdate, contributing to bacterial competition (*18*).

*A. baumannii*, among six leading antimicrobial-resistant pathogens (the “ESKAPE” organisms) globally threatening human health, is a notorious nosocomial pathogen, especially for immunocompromised patients (*19*–*21*). *A. baumannii* used a switch of virulent and avirulent by a master regulator and whose overexpression abrogated virulence and host innate immune antimicrobials including H_2_O_2_ play roles during murine infection of *A. baumannii* (*22*). Micronutrient acquisition systems are a key virulence mechanism, with previous studies mainly focusing on iron and zinc acquisition (*20*, *23*, *24*). Thriving in aerobic environment, *A. baumannii* primarily obtains Fe (III) by secreting siderophores under Fur transcriptional regulation (*25*). Then, the TonB-dependent receptor BauA transports the iron-siderophore complex into the periplasm (*26*), BauB and BauCDE complete delivery of the complex into the cytoplasm (*27*), and BauF reduces the iron-siderophore complex and releases Fe (II) (*28*). BasE, a blocking enzyme, disrupts siderophore biosynthesis as a regulatory control (*29*). The uptake and release of histidine-bound zinc (His-Zn) complexes are accomplished by the histidine utilization (Hut) system (*30*). Molybdate, iron and zinc trafficking, and MoCo biosynthesis share possible common regulators, forming a complicated network of interplaying actors (*5*).

We herein describe the specific molybdate binding protein *modA* in *A. baumannii* ATCC 19606. We perform a functional analysis of *modA* in bacterial physiology, including its impact on growth, morphology, and reactive oxygen species (ROS) tolerance. We also carried out a transcriptome analysis to study the role of ModA the regulation of molybdate, ferric ion, and zinc ion acquisition. Last, we have also solved crystal structures of ModA coordinated with molybdate or tungstate in reduced and oxidized states. The protein structure demonstrates a noncanonical disulfide bond and a redox-sensitive conformational change in ModA that differentially affects its binding affinity to molybdate versus tungstate. Our work provides important insights into a potential target for antimicrobial drug discovery.

## RESULTS

### *modA* deficiency leads to abnormal morphology and attenuated ROS tolerance in *A. baumannii*

**Fig. 1.**
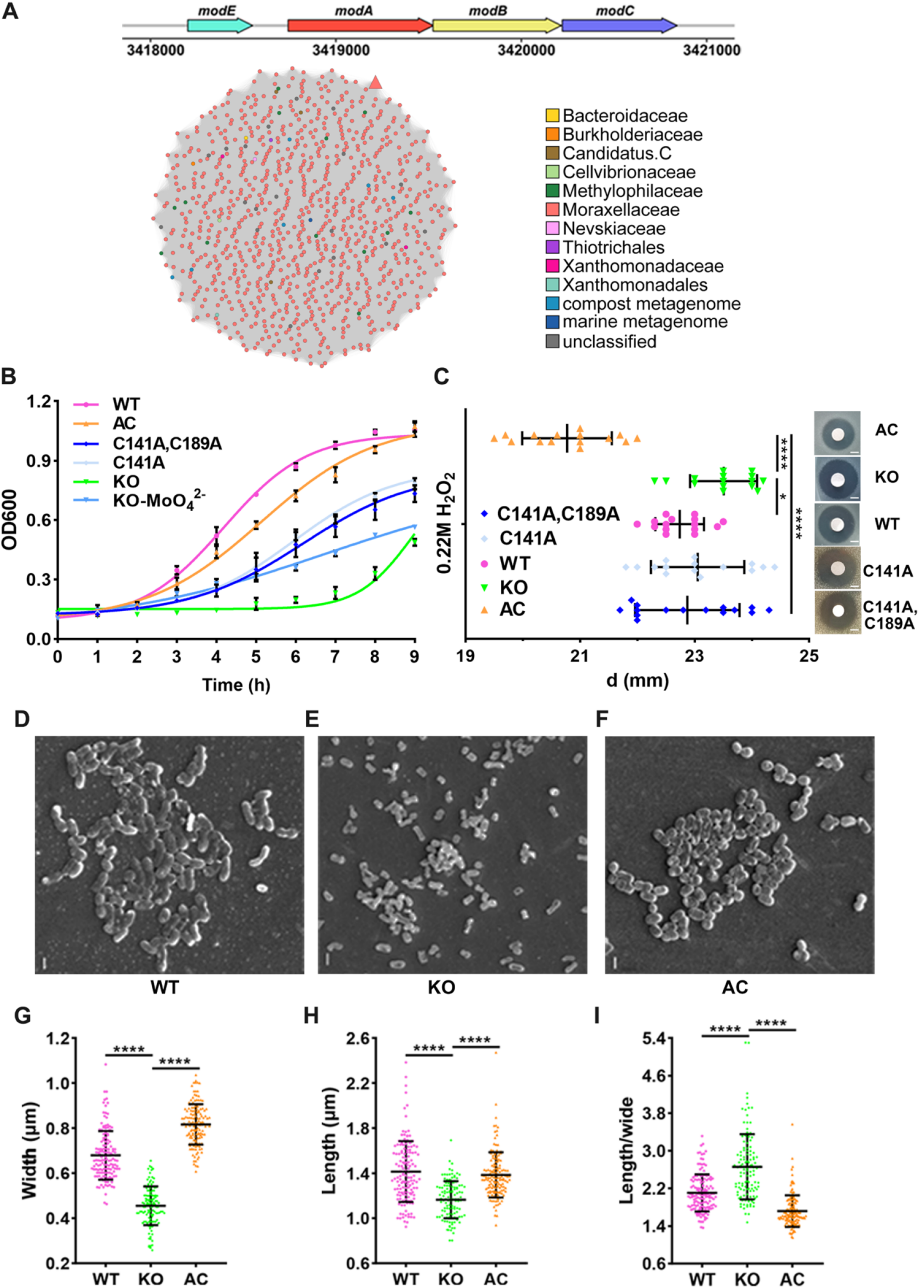
*modA* deficiency leads to abnormal morphology and attenuated ROS tolerance in *Acinetobacter baumannii*. (**A**) Sketch map of *modABC* operon in *A. baumannii* ATCC 19606 consisting of *modE*, *modA*, *modB*, and *modC*. Colors of the nodes represent the family from which the sequences originate. (**B**) Cultures of *A. baumannii* ATCC 19606 (WT), *modA* knockout strain (KO), *modA* complementary strain (AC), C141A complementary strain, and C141AC189A complementary strain KO shown significant growth defect compared to other strains and molybdate supplement (0.1 μg/ml) improved the growth to some extent. Values represent means ± SEs of three independent biological replicates. (**C**) KO shows significantly larger inhibition zone diameter compared to WT and AC after culture under H_2_O_2_, while no significant difference was harvest between C141A or C141AC189A and WT or KO. Images were selectively shown from three independent experiments; scale bars, 5 mm. One-way analysis of variance (ANOVA) was performed to calculate the statistical difference in GraphPad Prism, with *P* = 0.0392 and *P* < 0.0001 for WT and AC compared to KO, respectively. *P < 0.05; **P < 0.01; ****P < 0.0001. (**D** to **F**) Scanning electron microscope (SEM) observation of KO, AC, and WT strains, respectively, revealing smaller size, withered morphology, and poor adhesion to each other of KO cells compared to WT, with AC recovered certain degree to WT. (**G** to **I**) Statistical analysis of cell size in SEM pictures shows that the width and length of KO cells are significantly smaller than WT or AC strain; the ratio of length to width of KO increased significantly compared to WT or AC strains. ****P < 0.0001, calculated by one-way ANOVA in GraphPad Prism after three independent biological replicates with KO to WT or AC all *P* < 0.0001. Images are selective shown from three independent biological replicates; scale bars, 1 μm. h, hours.

The *mod* operon consisting of *modE*, *modA*, *modB*, and *modC*, was clustered, encoding the ModABC transporter responsible for molybdate uptake of *A. baumannii* ATCC 19606 (Fig. 1A and table S1). The open reading frame of *A. baumannii modA* is 699 base pairs (bp), encoding a 232–amino acid protein with GenBank accession number WP_000253147.1. ModA is an ancient molybdenum-sensing protein, and to illustrate the evolutionary locus of AbModA, we constructed sequence similarity networks. We performed PSI-BLAST searches using nonredundant protein and metagenomic bacterial protein databases. A total of 1149 similar protein sequences were generated with over 55% sequence identity to AbModA, covering 10 orders and bacteria from compost metagenome, marine metagenome, and unclassified others, among which Moraxellaceae shows greatly majority with 94.78%. Further AbModA presented divergent compared to other species in the clustered sequence similarity network of 970 nodes and 469,965 edges with 100% edge percentage identity, as located at the outside edge far from the central (Fig. 1A). The result indicated the relatively diversity of ModA in *A. baumannii*.

To investigate the function of the protein in bacterial physiology, we constructed a knockout strain of ModA (KO) following previously reported methods with modification (*31*). *modA* expression is significantly higher in the complemented strain of ModA (AC) than wild type (WT), whereas no expression is detected in the KO strain (fig. S1A). Several aspects of bacterial physiology were analyzed; first, KO of ModA resulted in decreased growth rate and lower density of stationary phase compared to WT, especially after 6 hours culture. Complementation of ModA (AC) can restore the growth at the early stage (Fig. 1B). Supplementation of molybdate (0.1 μg/ml) in the KO improved but could not fully restore the growth of the *A. baumannii*, indicating that other secondary uptake pathways may compensate, but ModA has a unique importance (Fig. 1B). The *modA*-deficient strain demonstrated decreased H_2_O_2_ tolerance, with the largest disk diffusion inhibition zone, whereas ModA complement strains showed a substantially smaller area compared to WT (Fig. 1C). Last, obvious morphology changes were detected using scanning electron microscopy, with ModA KO cells showing small, thin, and wizened rod-like shapes compared to WT or ModA complement strains (Fig. 1, D to F), with decreases in both width (Fig. 1G) and length (Fig. 1H) and higher length-width ratios (Fig. 1I) by statistics. The results suggest a role for ModA in bacterial growth, shape determination, and oxidative resistance in *A. baumannii*.

### *modA* affects metal ion homeostasis and multiple metabolic pathways

**Fig. 2.**
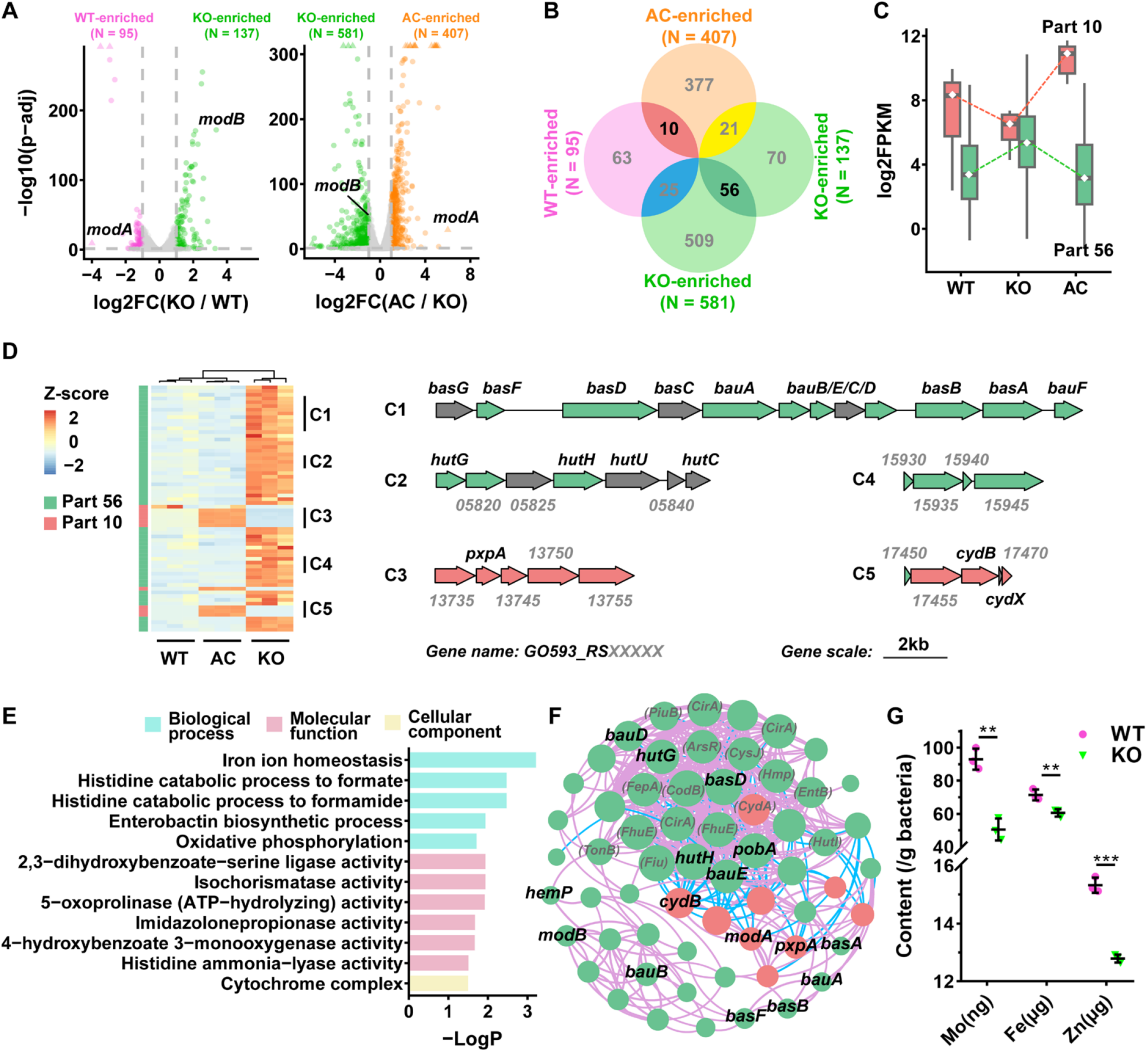
*modA* affects metal ion homeostasis and multiple metabolic pathways. (**A**) Volcano plot of DEGs in KO strain versus WT and AC by RNA sequencing. Significant DEGs are identified according to adjust *P* value (*p*-adj) < 0.05, and |log2 fold change (FC)| > 1. From *modABC* operon, *modB* shows significantly up-regulated after *modA* knockout. *x* axis represents log^2^ FC, *y* axis represents −log10 (*p*-adj). (**B**) DEGs analysis generates Venn diagram to identify 10 KO enriched DEGs (part 10) marked as orange and 56 WT and AC both enriched DEGs (part 56) labeled as green. (**C**) The expressed pattern of part 10 and part 56 DEGs. Part 10 shows decreased tendency after ModA deletion and recovers in complement strain; on the contrary, part 56 DEGs increase the transcription after ModA knockout and drop back in complement strain. (**D**) Heatmap of part 10 and part 56 DEGs by hierarchical clustering. Colors from blue to orange, indicates the increasing expression level based on *Z* score. The number of continuous genes at least three was marked as cluster (shown as C in the picture). (**E**) Top 12 rich distribution of DEGs in GO term assay of part 10 and part 56 together. (**F**) Correlation of DEGs expression network of part 10 (orange) and part 56 (green) together. The pink and blue lines indicate gene’s positive and negative interactions, respectively. The size of node indicates the magnitude of the association in the network. (**G**) The content of molybdenum, iron, and zinc decreased significantly after *modA* knockout compared to WT. Unpaired Student’s *t* test was performed with WT compared to KO for each metal ion, with *P* = 0.0039, *P* = 0.0011, and *P* = 0.0009 for Mo(ng), Fe(μg), and Zn(μg), respectively. **P < 0.01; ***P < 0.001.

To explore the mechanism of ModA in growth, shape determination, and oxidative tolerance, we carried out transcriptomics analysis of WT, KO, and AC (ModA complementary) at optical density at 600 nm (OD_600_) = 0.6. The differentially expressed genes (DEGs) were analyzed by two groups as KO versus WT and AC versus KO. A total of 95 significantly down-regulated DEGs and 137 up-regulated DEGs were identified with *modA* gene knockout in KO compared to WT, while 407 DEGs and 581 DEGs showed significantly up-regulated and down-regulated with *modA* gene complemented in AC compared to KO, respectively. After *modA* knockout, expression of *modB* in *mod* operon increased (Fig. 2A). To validate the genes influenced by *modA’*s presence and absence, we performed an intersection of the two groups KO versus WT and AC versus KO, 10 down-regulated DEGs in *modA* knockout strain were shown through intersection of WT and AC enriched DEGs (part 10), and 56 up-regulated DEGs in *modA* deficiency strain (part 56, Fig. 2B). The expression pattern of DEGs in part 10 decreased with *modA* knockout, but increased in *modA* complementary strains. On the contrary, the expression pattern of DEGs in part 56 increased after *modA* gene knockout and decreased with *modA* gene complementation (Fig. 2C). Five clusters were identified from intersecting parts containing at least three continuous genes, including *bauA*, *hutH*, *pxpA*, *RS15935 (piuB)*, *cydB*, and their neighboring genes, among which three clusters related to metal acquisition. *bauA* and *PiuB* are associated with iron uptake and homeostasis, while *HutH* is responsible for zinc acquisition. Two clusters down-regulated by *modA* knockout are *pxpA* and *cydB*, involved, respectively, in the degradation of 5-oxoproline (OP) to glutamate (*32*, *33*) and the constitution of cytochrome bd-I (CydAB) for aerobic respiration (*34*), (Fig. 2D). Gene ontology (GO) term assay showed that the up-regulated DEGs in part 56 were enriched in iron ion homeostasis, zinc uptake through Hut system, oxidative phosphorylation, and isochorismatase activity. The significantly up-regulated isochorismatase (RS16395) confers increased siderophore-mediated ferric iron acquisition ability, and it was previously identified to be a major factor involved in the recognition of intracytosolic *A. baumannii* and the induction of autophagy (*35*). Enterobactin biosynthetic is included into the iron scavenge using siderophores. While two operon genes neighboring *pxpA* and *cydB* are included in part 10, further enriched as 5-oxoprolinase (OPLAH) activity (*32*, *33*) and cytochrome complex, respectively (Fig. 2E). The consistent results were observed in the Kyoto Encyclopedia of Genes and Genomes (KEGG) pathway, including histidine metabolism, siderophore group peptide biosynthesis, and oxidative phosphorylation (fig. S1B). Stronger correlations were detected among iron homeostasis throughout the gene network of DEGs by *modA* deficiency, with 8 of top 12 DEGs are iron transport and metabolism related genes. The correlation of *modA* to other fluctuated genes is higher than *modB* (Fig. 2F and fig. S1C). To verify the impact on metal uptake, we conducted a metal content assay by inductively coupled plasma mass spectrometry (ICP-MS) and found that the cytoplasmic content of molybdate, iron, and zinc were significantly decreased after *modA* knockout (Fig. 2G).

### ModA contains a redox-sensitive noncanonical disulfide bond, modulating molybdate binding affinity

**Fig. 3.**
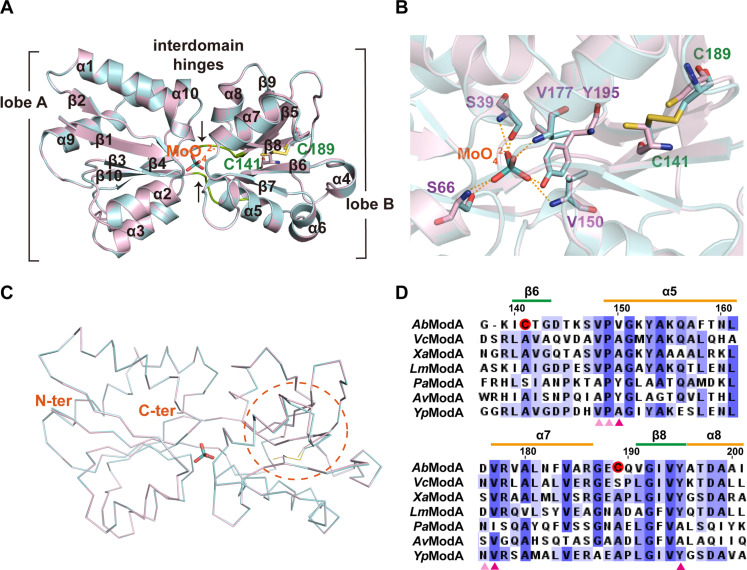
ModA uses a noncanonical disulfide bond interplay with ROS, modulating molybdate sensing affinity. (**A**) Superposition of ModA structure occupied by a molybdate in oxidized and reduced state in cartoon presentation. Oxidized and reduced ModA is shown in cyan and light pink, respectively, while MoO_4_^2−^ is shown as stick. (**B**) Superposition of metal-binding pocket of molybdate-bound ModA in oxidized and reduced state is shown in ribbon representation. Residues interacting with the ligands are shown in stick representation and bonds between the structure and molybdate are shown by dashed lines. The hydro sulphonyl and disulfide bond from Cys^141^ and Cys^189^ are shown in yellow. (**C**) Superposition of molybdate-bound ModA in oxidized and reduced state is shown in line representation. The changed conformation position between two states is circled by dashed lines. N-term and C-term labeled as the N and C termini, respectively. (**D**) Sequence alignment around two novel cysteines of ModA in *A. baumannii* ATCC 19606 to other top6 structural similar ModAs. Cys is marked by red circle, and directly and indirectly interacting residues with molybdate are labeled by pink triangle and orange triangle, respectively. The secondary structure α helix and β sheet is marked by orange and green lines, respectively. The abbreviations *Vc*ModA, *Xa*ModA, *Lm*ModA, *Pa*ModA, *Av*ModA, and *Yp*ModA represents 4RXL (*Vibrio cholerae* O1 biovar El Tor str. N16961), 2H5Y (*X. citri* pv. *citri* str. 306), 7TAV (*Listeria monocytogenes*), 7T5A (*P. aeruginosa* PA1), 1ATG (*Azotobacter vinelandii*), and 6NIO (*Yersinia pestis*), respectively.

**Fig. 4.**
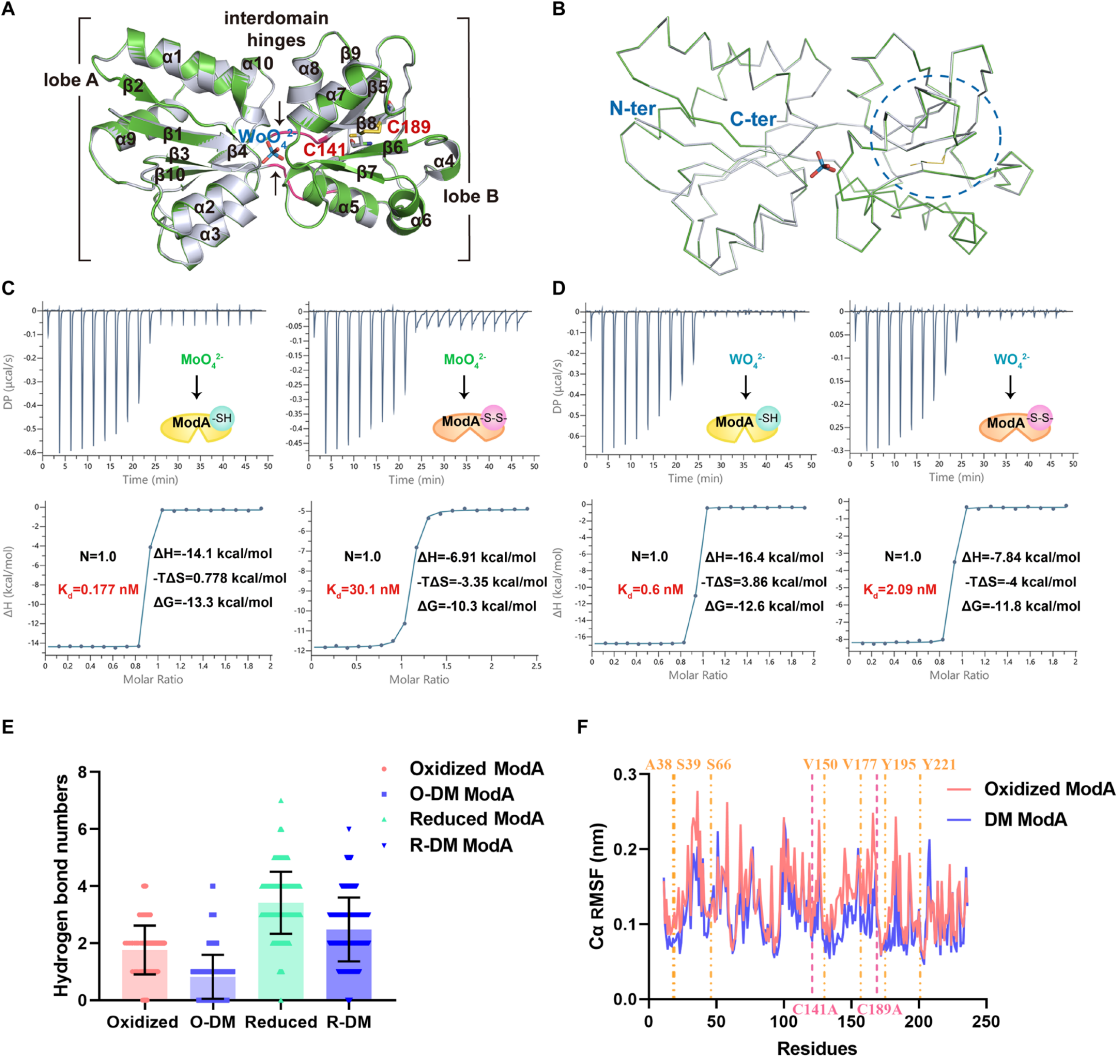
The noncanonical disulfide bond of ModA in *A. baumannii* ATCC 19606 is associated with molybdate sensing affinity. (**A**) Superposition of ModA structure occupied by a tungstate in oxidized and reduced state in cartoon presentation. Oxidized and reduced ModA is shown in tv_green and blue white, respectively, while MoO_4_^2−^ is shown as stick. (**B**) Superposition of tungstate-bound ModA in oxidized and reduced state is shown in line representation. The changed conformation position between two states is circled by dashed lines. N-term and C-term labeled as the N and C termini, respectively. (**C**) Isothermal titration calorimetry (ITC) curves of ModA in oxidative (containing -S-S-) and reduced (containing -SH) state against molybdate. The typical data were presented with three repeats performed. (**D**) Isothermal titration calorimetry (ITC) curves of ModA in oxidative (containing -S-S-) and reduced (containing -SH) state against tungstate. The typical data were presented with three repeats performed. (**E**) Hydrogen bonds to molybdate in ModA by MD simulation. O represents oxidative state, DTT for reduced state, and AA stands for ModA with C141A and C189A double mutant (DM). (**F**) Cα RMS fluctuation of oxidative ModA compared to ModA with C141A and C189A double mutant generated by MD simulation.

We expressed and purified recombinant AbModA, which eluted as a monomer on size exclusion chromatography (fig. S2A). We crystallized ModA in both oxidized and reduced states bound with molybdate or tungstate, and the structures were solved at atomic resolution of 1.44, 1.39, 1.44, and 1.35 Å, respectively (table S2). The x-ray diffraction data collection and relevant statistics are summarized in table S2. The overall structure of ModA bound to molybdate or tungstate assembles with two lobes, lobe A and lobe B, connected by interdomain hinges (Figs. 3A and 4A). ModA contains 10 α helices and 10 β sheets; lobe A consists of two noncontinuous parts β1α1β2α2β3α3β4 and β10α9α10, while lobe B consists of β5α4β6α5α6β7α7β8α8β9 (Figs. 3A and 4A), which indicates that AbModA is a type II substrate-binding protein (*11*). The MoO_4_^2−^ or WO_4_^2−^ is chelated at the center between the two lobes with contribution from the interdomain hinges (Figs. 3A and 4A). The oxygen atoms of MoO_4_^2−^ or WO_4_^2−^ are hydrogen bonded to the backbone N-H groups of S39, S66, V150, V177, and the side chain hydroxyl of Y195 (Fig. 3B and fig. S2B). Upon oxidization with peroxide, a disulfide bond between C141 and C189 in MoO_4_^2−^ or WO_4_^2−^ liganded complexes was detected (Fig. 3B and fig. S2B), inducing a conformation change of α5α7β8α8 of lobe B (Figs. 3, C and D, and 4B). Dali search queried by AbModA returned six structures, with the sequence and structure alignments within the ModA family demonstrating two noncanonical cysteines (C141 and C189) unique to *A. baumannii* and not found in other bacteria (Fig. 3D and fig. S2, C and D). To investigate the impact of disulfide bond formation of substrate binding, we performed isothermal titration calorimetry (ITC) experiments to characterize the interaction between MoO_4_^2−^ and ModA and found that the affinity of ModA for MoO_4_^2−^ decreased from 0.177 to 30 nM upon oxidation (Fig. 4C).

As group VI metal oxyanions close to molybdate in the periodic table, tungstate and chromate were compared to molybdate in a heat shift assay. The heat stability increased significantly with 5 μM MoO_4_^2−^ supplementation (fig. S3A), whereas the other oxyanions required higher concentrations: 20 μM for WO_4_^2−^ (fig. S3B) and 80 μM for CrO_4_^2−^ (fig. S3C). We were successful in obtaining crystals for ModA in complex with MoO_4_^2−^ or WO_4_^2−^, but not with CrO_4_^2−^. Consistent with the higher heat stability of ModA bound to MoO_4_^2−^ than WO_4_^2−^, higher energies of ModA bound with molybdate than tungstate in oxidized or reduced state were observed from crystallization (fig. S3D). The conformational change within the α5α7β8α8 between the reduced and oxidized ModA was slightly significant in MoO_4_^2−^ compared to WO_4_^2−^ (Figs. 3C and 4B. The affinity of ModA for WO_4_^2−^ dropped from 0.6 to 2.09 nM upon oxidation, a miniscule change compared to the drop observed with MoO_4_^2−^ (Fig. 4, A and B). No interaction was detected between ModA and SO_4_^2−^ (fig. S3E) or PO_4_^2−^ (fig. S3F).

To further explore the physiological function of the disulfide bond, we generated alanine substitution mutants for Cys^141^ and Cys^189^. Complementation by either double substitution (C141A and C189A) or single substitution (C141A) restored bacterial growth to some extent (Fig. 1B), while the inhibition zones of the mutant complement strains show no significant difference to WT or KO (Fig. 1C). Complementation with the mutants did not markedly decrease the inhibition zone in the manner of complementation with WT ModA. For the double C141A and C189A mutant (fig. S4A) and the single C141A mutant (fig. S4B), the affinity of ModA for MoO_4_^2−^ increased compared to oxidized form of ModA.

The total numbers of hydrogen bonds were more in reduced state than oxidative state of ModA, and the C141A and C189A double mutation decreased the total number of hydrogen bonds to molybdate as shown by molecular dynamics (MD) simulation (Fig. 4E). Cα RMS fluctuations in the key region A39-S66, V150-V177, and Y195-Y211 involved in MoO_4_^2−^ binding of the oxidized WT ModA structure (Fig. 4F) indicate that it is more dynamic than the double mutant.

### *modA* deficiency in *A. baumannii* ATCC 19606 reduced virulence in murine pneumonia model

**Fig. 5.**
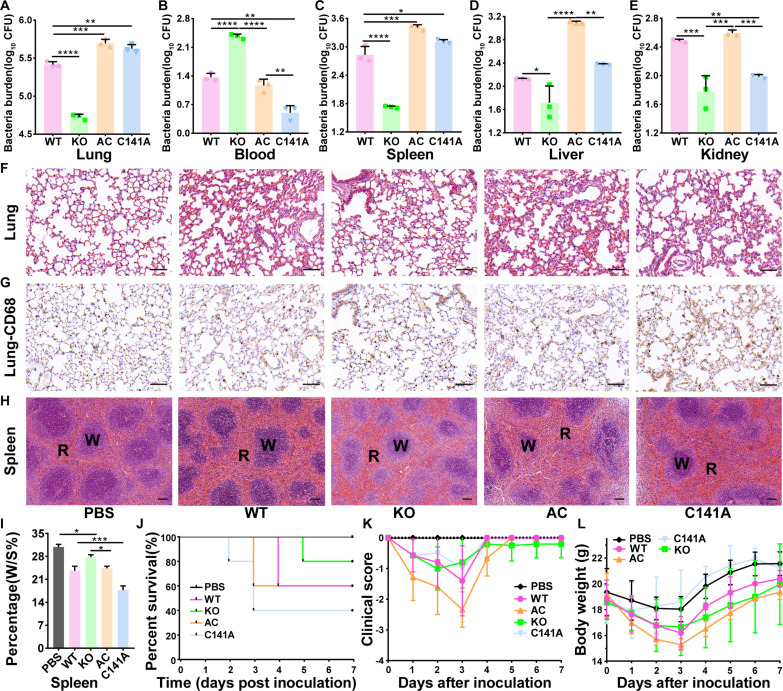
*modA* deficiency in *A. baumannii* ATCC 19606 reduced virulence to mice nasal feeding infection model. (**A** to **E**) *A. baumannii* load in the lungs, blood, spleens, liver, and kidney of the mice after intranasal inoculation with WT, KO, AC, and C141A, respectively. In lungs, spleen, liver, and kidney, the CFU of KO is significantly lower to other group while in blood, the CFU of KO is significantly higher, with all *P* value < 0.0001. All statistics significances were calculated by one-way ANOVA. (**F** to **H**) Histopathological analysis of the lung and spleen tissues infected mice. Inflammation infiltrates area of inflammation and inflammatory cell counts of KO reduced compared to WT, AC. The macrophages marked by CD68 as brown assembled after bacterial invasion, especially more in WT, AC treatment compared to KO and PBS. The area of red pulp relative to white pulp labeled by W and R, respectively, increased after bacterial infection. All images are displayed with scale bars = 100 μm. (**I**) The percentage of area of white pulp to whole spleen decreased significantly after *A. baumannii* infection. The ratio of KO is significantly higher to WT, AC and C141A with *P* value = 0.0036, *P* value = 0.0137, and *P* value < 0.0001, respectively. All statistics significances were calculated by one-way ANOVA on GraphPad Prism. (**J**) Survival curve of mice nasal feeding infection model. (**K**) The clinical score shows day 0 (before inoculation) last to day 7. From day 0 to day 1, day 2 to day 3 clinical score of infected mice dropped faster. KO infected mice recover from day 2, while WT, AC and C141A group reaches the lowest clinical score at day 3. (**L**) The body weight shows day 0 (before inoculation) last to day 7. The body weight decreased fastest from day 0 to day 1. The AC group appears the lightest weight among three groups at day 3.

To better understand the role of *modA* in pathogenicity, we investigated the inoculation of *A. baumannii* in the lungs of mice with cyclophosphamide-induced neutropenia. Considering the similar phenotype observed of C141A with C189A and C141AC189A double mutants in bacterial growth curve and H_2_O_2_ tolerance, C141A strain has been used for the murine pneumonia model investigation. One day after nasal inoculation of fresh bacterial strains of WT, KO, AC, and C141A, respectively, three mice of each group were euthanized for quantitative bacteriology and histopathology assays. The bacterial burden of *modA* knockout strain in lung (Fig. 5A), spleen (Fig. 5C), liver (Fig. 5D), and kidney (Fig. 5E) was significant reduced, although greater numbers were found in blood (Fig. 5B), compared to WT or complemented strains. Under histopathologic observation of lung tissue, the inflammatory infiltrate was less severe in *modA* knockout, with smaller areas of inflammation, lower numbers of inflammatory cells, and less edema of the interstitial spaces (Fig. 5F). CD68 staining indicated greater infiltration of macrophages after infection of WT or complement strains than *modA* knockout strain (Fig. 5G) (*36*). The area of white pulp in the spleen is reduced after *A. baumannii* infection (Fig. 5H), with the ratio of area of white pulp to whole spleen significantly higher in the knockout strain than WT and complemented strains (Fig. 5I). No significant tissue damage was observed in liver (fig. S5A) or kidney (fig. S5B). In terms of survival, the infection of KO was mild with only one mouse dead at day 5, while two mice died from the AC and WT infected groups at day 3 and day 4, respectively. Three mice died by day 3 in the C141A-ModA infection group (Fig. 5J). Consistent with the bacterial burden and histological assays, the KO strain showed decreased virulence. The KO-inoculated mice group was the earliest (day 2) to recover as indicated by clinical score compared to WT or complement strains’ infected groups (Fig. 5K), although this was not completely reflected in body weight recovery (Fig. 5L). In conclusion, ModA is involved in the pathogenicity of *A. baumannii*, with decreased bacterial burden, attenuated tissue damage, and improved clinical score caused by *modA* knockout.

## DISCUSSION

The early appearance and conservation of the molybdopterin synthesis pathway in the course of evolution stresses the importance of molybdate in metabolism (*14*). Molybdenum-containing enzymes are present in all life forms, whereas tungsten-containing enzymes occur typically in thermophilic obligate anaerobes, although some mesophilic anaerobes are transitional in that they can use either molybdenum- or tungsten-containing enzymes depending on availability and growth conditions. Elevated intracellular redox poise of aerobic organisms may be an explanation of preference (*14*). However, tungsten can replace molybdenum in MoCo when tungsten is in excess, which inhibits the activities of bacterial molybdoenzymes (*37*–*39*).

As a trace element, molybdate requires high affinity capture by ModA for bacterial pathogenicity. In humans, the homolog of MOT2 has been detected and showed an apparent Michaelis-Menten constant (Km) of about 550 nM (*40*), while the affinity in bacteria is much higher. ModA of *Klebsiella pneumoniae*, *P. aeruginosa*, *Bacillus*, *P. fluorescens*, and *E. coli* bind to molybdate with dissociation equilibrium constant (K_D_) values of 6.3, 1.0, 2.2, 27.0, and 25.0 nM, respectively (*16*, *41*, *42*). In *A. baumannii*, ModA has a K_D_ value of 0.177 nM to molybdate, which is significantly higher than the host or competing bacteria. The mammalian host imposes nutritional scarcity by regulating trafficking or releasing high-affinity metal-chelating proteins to decrease availability of micronutrients in response to invading pathogens. Considering the low affinity of reported human Mot2 and the incompletely understood mechanism of molybdenum uptake and transport (*40*), there may exist other proteins or other strategies for the host to outcompete the invader in the scramble for molybdate (*1*, *43*).

In this study, we discovered a noncanonical disulfide bond in ModA of *A. baumannii*, which changes the affinity for substrates like molybdate and tungstate, a potential regulatory switch dependent on the environment.

**Fig. 6.**
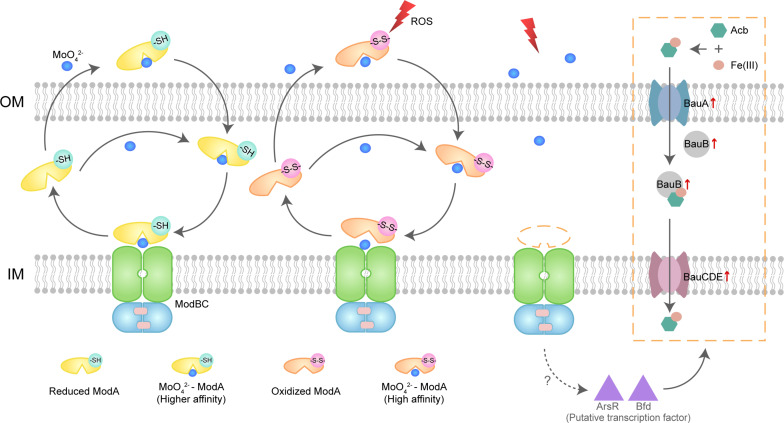
Schematic mechanism of ModA interplay with ROS. Molybdate uptake by ModA is modulated by a noncanonical disulfide formation. The reduced ModA demonstrated a higher affinity than the oxidized state to molybdate. The molybdate update by ModA is also correlated with the ferric ion uptake through the BauABCDE pathway.

ModA has evolved diverse types to adapt to specific habitat. ModA from archaeon *Methanosarcina acetivorans* uses octahedral coordination of molybdate, which is different from the more common tetrahedral coordination (*44*). A salt bridge between K127 and D59 of ModA from *Xanthomonas citri* is essential to maintain the structure and binding properties (*45*). Two lobes of ModA surround the substrate-binding site and are connected by a hinge that provides flexibility, allowing them to swing open in the absence of substrates (*8*). The formation of the ModABC complex in the absence of molybdate is unfavorable, while binding of molybdate triggers docking of ModA onto ModBC (*9*). In our study of *A. baumannii*, the formation of a disulfide bond causes conformational changes to lobe B of molybdate-bound ModA, but the impact of ROS oxidation on the ModABC complex needs further study. In the oxidizing environment of the periplasm, it is possible that ModA always exists in the oxidized form, although it is possible that due to some structural instability of the oxidized form, the redox potential of ModA in *A. baumannii* favors the reduced form, which contains more total hydrogen bond as shown by MD simulation and makes ModA a redox-sensitive signal sensor (*46*). The hypothesis has been also cross-validated that the C141A mutant and disulfide bond double-mutant phenotypes are more similar to WT than the KO in bacterial growth, H_2_O_2_ tolerance and murine infection experiment. We found that the oxidized form of ModA has a lower affinity for molybdate, but the higher degree of structural flexibility seen in MD simulations could help it to release molybdate to ModBC to facilitate uptake (Fig. 6).

ModA is linked to the uptake of metal ions, especially iron acquisition through Bau system by the regulators ArsR and Bfd (Fig. 6). The acquisition of molybdate, iron, and zinc ions exhibits a close interplay in bacteria, and the expression of RS15390 (*ArsR*) and RS04680 (*Bfd*) in *modA* knockout strain, two of three of the significantly up-regulated transcriptional regulators, may be the reason. ArsR is a global regulator protein associated in metal homeostasis and hostile environments resistance (*47*, *48*). Bfd is a cognate ferredoxin, functioning in regulation iron levels in complex with BfrB (*49*, *50*). Another regulator is AraC (RS16315), which was identified initially as the arabinose metabolic pathway regulator, but its orthologs have since proved to be involved in virulence, oxidative stress response, and the regulation of the expression of extracellular proteins (*51*–*53*).

OP is an inevitable by-product of glutamate metabolism, and OP disposal systems are common in all domains of life. OPLAH is an ATP-hydrolyzing enzyme involved in the conversion of the metabolite OP into glutamate. Prokaryotic OPLAH complex *pxpA*, *pxpB*, and *pxpC* genes occur in tandem (*33*). Inactivation of *Bacillus subtilis pxpA*, *pxpB*, or *pxpC* genes slowed growth, caused OP accumulation in cells and medium (*32*), so down-regulation of these genes is a potential explanation for the growth defect observed in the *modA* deletion strain.

Knockout of ModA has far reaching effects on gene expression, ROS tolerance, and virulence. Further work is needed to dissect out whether these effects are due to increased production and activity of various molybdate-containing oxidoreductase enzymes or due to other downstream metabolic effects as described above. Another possibility is the activation of a phenotypic switch, a master regulatory gene that controls the transition from an avirulent to a virulent phenotype, as has been described previously for *A. baumannii* (*22*). However, we note that *A. baumannii* is known for multiple antibiotic resistance genes and biofilm production, and we did not observe up-regulation of such genes suggestive of such a class switch. It is important to determine whether the impact of ModA on virulence is specific to molybdate uptake or on other downstream factors that are affected by multiple converging pathways. The former would make it a far more promising antibiotic target.

## MATERIALS AND METHODS

### Sequence similarity networks construction

The sequence of AbModA from *A. baumannii* ATCC 19606 was used as a query to conduct PSI-BLAST searches against the National Center for Biotechnology Information nonredundant protein sequence (nr) and metagenomic protein (env_nr) databases until convergence (*54*), resulting in 1149 protein sequences over 55% sequence identity. Signal peptides (SPs) of ModA sequences were predicted using SignalP (version 6.0) (*55*), and then manually curated for 44 SP missing sequences; SP was removed before further analysis of the sequences.

The similarity between all protein sequence pairs was calculated by the Enzyme Function Initiative-Enzyme Similarity Tool with an *E* value threshold of 1 × 10^−5^ (*56*). The resulting sequence similarity network of 970 nodes and 469,965 edges with 100% edge percentage identity was constructed, explored, and visualized using the organic layout through Cytoscape (version 3.9.1) (*57*).

### Cloning, expression, and purification of recombinant AbModA

AbModA knockout strain was constructed as previous description (*58*). Five hundred base pairs upstream and 500 bp downstream of *AbModA* and pKD4 plasmid kanamycin cassette in the middle replacing original *AbModA* were amplified by polymerase chain reaction (PCR) using the primers listed in table S3 with homologous fragment and then seamless cloned into pET28a vector. After the verification of DNA sequencing, the united PCR products of three parts without homologous flank were transformed into *A. baumannii* ATCC 19606 with pAT04 containing REC_AB_ by electroporation. The knockout strain was validated by ID primers listed in table S3.

The recombination vector of pET28a-AbModA for protein expression was built by double digest using Nco I and EcoR I, with *AbModA* fragment removed signal peptide amplified from *A baumannii* ATCC 19606 genomic DNA. Isopropyl β-d-1-thiogalactopyranoside (IPTG; 0.5 mM) was used to induce the expression of recombination protein in *E. coli* strain BL21 (DE3), when OD_600_ growing to 0.6 to 0.8. After culturing in 18°C for about 16 hours transformed from 37°C, the cell pellets were collected at 4°C by centrifugation at 6000*g* for 10 min. Buffer A (20 mM tris, 150 mM NaCl, and 5% glycerol) was used to resuspend the cell pellet and through ultrasonication. The lysate was loaded to an Ni–nitrilotriacetic acid agarose column (Qiagen) after removing cell debris, equilibrated with buffer A, washed with buffer A carrying 25 mM imidazole, and eluted with buffer B (20 mM tris, 150 mM NaCl, 5% glycerol, and 500 mM imidazole). Then buffer A was still pre-equilibrated in a Superdex 75pg, Hiload 16/60 (GE Healthcare Life Sciences) size exclusion chromatography column for further purification. All protein purification steps were performed at 4°C.

To prepare oxidized ModA, WT ModA was treated with a fivefold molar excess of H_2_O_2_, incubated at 25°C for 3 min, and quickly desalted using a Hitrap desalting column (GE Healthcare), equilibrated in 20 mM tris-HCl (pH 8.0) and 150 mM NaCl. Reduced ModA was produced by adding 0.5 mM dithiothreitol (DTT) to WT ModA (*59*). The oxidized and reduced state proteins were promptly purified using size exclusion chromatography and then used for isothermal titration calorimetry and crystallization trials.

### Scanning electron microscopy

The ultrastructure of *A. baumannii* was observed by scanning electron microscopy. Overnight cultured bacteria were diluted to OD_600_ = 0.1, then transferred 1 ml into a chamber in 12-way plate each soaking with a sterile slide, and cultured still at 37°C for 24 hours. Four percent paraformaldehyde was used to firstly fix cells for 20 min at room temperature, and then 2.5% glutaraldehyde was performed at 4°C overnight. Slides were washed four times by phosphate buffer, each time for 15 min. Ethanol dehydration with increasing gradient from 30, 50, 70, 80, to 90%, each step for 15 min. At last, slides were treated with 100% ethanol for 30 min and three times. After sputter-coating with gold, the samples were observed under MAIA3LMH (TESCAN, Czech Republic). ImageJ was used to calculate the length and width, statistical analysis was performed on GraphPad with three independent biological replicates of experiment.

### *A.baumannii* mouse infection model

Six-week-old female C57BL/6JNifdc mice were raised in the same condition during 1-week adaptation and experiment. Cyclophosphamide (150 mg/kg) was used twice to pretreat mice at day 4 and day 1 to induce mice neutropenic before bacterial inoculation as described (*60*). For intranasal inoculation, *A. baumannii* strains were cultured overnight, washed three times with saline, and then diluted to appropriate optical density value. Approximately 1 × 10^8^ colony-forming units (CFU) of bacteria in 50 μl of saline were intranasally inoculated to mice under anesthesia by isoflurane. The clinical symptoms were monitored and scored. One day after inoculation, three infected mice from each group were euthanized, blood samples were collected for quantitative bacteriology, and lungs, spleens, liver, and kidney; the relevant organs were harvested for further quantitative bacteriology or histopathology assay.

### Bacterial count (CFU) and histopathological assay

Homogenizers (MiNiBeadbeater-16, BioSpec Products Inc. USA) were used to perform the homogeneity of the organs with 500 μl of sterile saline. The numbers of infected *A. baumannii* in the organs and blood samples were quantified through 10-fold serial dilutions on LB agar plates cultured overnight.

For histopathology observation, the specimens were demonstrated standard paraffin embedding methods after 4% paraformaldehyde fixing upon harvested immediately. The paraffin blocks were sliced and stained by hematoxylin and eosin; last, the tissue slices were examined by fully automatic digital slicing scanner (Pannoramic DESK, 3DHISTECH, Hungary).

### Thermal shift assay of AbModA occupied to oxyanions

Purified recombinant AbModA (10 μM) was incubated with Na_2_MoO_4_, Na_2_WO_4_, and Na_2_CrO_4_ at series concentrations ranging from 0 to 80 μM, respectively, in buffer A for 30 min before running on the program. A protein thermal shift dye kit (Thermo Fisher Scientific Applied Biosystems) was used to detect fluorescence intensity with the program of an increase of temperature from 25° to 98°C with 0.1°C step size on a Bio-Rad CFX96 C1000 Touch Thermal Cycler, as described previously (*58*). Three independent biological replicates were performed, and data were analyzed on GraphPad Prism (La Jolla, CA, USA).

### Isothermal titration calorimetry analysis of AbModA binding to oxyanions

The AbModA purified recombinant AbModA, together with Na_2_MoO_4_, Na_2_WO_4_, Na_2_SO_4_, and Na_3_PO_4_ were diluted to 20 and 200 μM, respectively, using the same stock solution which was the buffer A equilibrating the size exclusion chromatography column during the protein purification. ITC assay was conducted on a MicroCal PEAQ-ITC (Malvern) at 25°C as previous reported (*61*). One hundred microliters of oxyanions were titrated into the sample chamber containing 300 μl of AbModA. Data were analyzed through MicroCal PEAQ-ITC analysis software (Malvern). Three independent biological replicates were performed.

### Crystallization

The 384-well plates were used to conduct initial crystallization screening experiments by sitting drop vapor diffusion method at 20°C, using commercially available sparse matrix screen kits (Molecular Dimensions and Hampton Research). Protein solution mixes (0.5 μl) with 0.5 μl of reservoir solution equilibrating against the same 45 μl of reservoir solution. The concentration of AbModA is 18 mg/ml for crystallization, and all crystals were formed in the solution of CSI + II F1, with 0.1 M sodium acetate trihydrate (pH 4.6) and 30% polyethylene glycol monomethyl ether 2000.

### Data collection and structure determination

We collected datasets at Shanghai Synchrotron Radiation Facility Beamline 18U1 and 19U1, and then processed and scaled the x-ray diffraction data on XDS (*62*). Data collection details and statistics are summarized in table S2. The Phaser program suite was implemented with maximum-likelihood molecular replacement to determine the structure of AbModA with 2H5Y as search model sharing 46% identity to AbModA. PHENIX and COOT suite were used to perform manual model rebuilding and refinement (*63*, *64*). Last, the crystal structure of reduced AbModA in complex with MoO_4_^2−^ was solved at 1.39 Å in space group *C*2 with cell dimensions *a* = 85.6, *b* = 39.9, and *c* = 79.3 Å and α = 90, β = 109.4, and γ = 90; the structure was refined to a final Rwork/Rfree of 0.1620/0.1932. The crystal structure of oxidized AbModA in complex with MoO_4_^2−^ was solved at 1.44 Å in space group *P*1 with cell dimensions *a* = 47.3, *b* = 73.8, and *c* = 79.1 Å and α = 97.7, β = 107.4, and γ = 100.6; the structure was refined to a final Rwork/Rfree of 0.2561/0.2755 because of slightly twining issue. The crystal structure of reduced AbModA in complex with WO_4_^2−^ was solved at 1.35 Å in space group *C*2 with cell dimensions *a* = 85.6, *b* = 40.0, and *c* = 79.3 Å and α = 90, β = 109.5, and γ = 90; the structure was refined to a final Rwork/Rfree of 0.1667/0.1946. The crystal structure of oxidized AbModA in complex with WO_4_^2−^ were solved at1.44 Å in space group *C*2 with cell dimensions *a* = 86.1, *b* = 39.7, and *c* = 79.4 Å and α = 90, β = 109.3, and γ = 90; the structure was refined to a final Rwork/Rfree of 0.1768/0.1860. All structure illustrations were generated through the program Pymol version 1.8, Schrödinger.

### H_2_O_2_ tolerance assay

A disc diffusion assay was used to detect H_2_O_2_ resistance (*59*). After overnight culture, the bacteria were diluted to OD_600_ = 0.3 and inoculated into cooled melting solid LB with 2 mM IPTG and proper antibiotic. Fifty milliliters of LB was poured into three petri dishes, and then the same size sterile circle filter paper containing 10 μl of 0.22 M H_2_O_2_ was place on the surface. The diameter of inhibition zone was measured after overnight culture in 37°C. Three biological repeats were performed and data were analyzed in GraphPad Prism.

### RNA sequencing and analysis

Single colony of WT, KO, and AbModA was cultured to OD_600_ = 0.6, respectively. Then, TRIzol Reagent/RNeasy Mini Kit (Qiagen) was used to prepare total RNA of strain samples. The usage of Agilent 2100/2200 Bioanalyzer (Agilent Technologies, Palo Alto, CA, USA), NanoDrop (Thermo Fisher Scientific Inc.), and 1% agarose gel was to quantify and qualify the total RNA. A next-generation cDNA library was constructed by 1 μg of total RNA after the treatment of rRNA removal kit following the manufacturer’s protocol. An Illumina HiSeq loaded the libraries to generate a high sequencing depth of more than 14.5 million reads per sample. The raw reads were filtered to remove the adaptors and low-quality bases using cutadapt (version 1.9.1) (*65*) and then were mapped to the *A. baumannii* strain ATCC 19606 chromosome, complete genome (accession CP046654) through bowtie2 (version 2.2.6).

DEGSeq (version 1.38.0) (*66*) was used to calculate differential expression, significant differential expression selected on the basis of two criteria:∣log2 fold change∣ > 1 and adjusted *P* value (*p*-adj) < 0.05. Gene expression pattern was clustered by hierarchical clustering using *Z* score normalized gene count. KEGG pathway conforming to two requirements: the number of DEGs >3 and *P* value < 0.05 was distributed by the rich factor. Gephi version 0.9.2 visualized correlations of gene expressing networks with absolute correlations above 0.6 and *P* value < 0.001.

### MD simulation

The structures of oxidized ModA and double-mutant ModA with MoO_4_^2−^ were used in MD simulation study. To simulate the ModA_C141A_C189A mutant, the cysteine acids were replaced with alanine using the COOT program (*64*). The Sobtop software was used to calculate the stereochemical properties of the molybdate ligand [Tian Lu, Sobtop, Version [1.0(dev4)], http://sobereva.com/soft/Sobtop (accessed on 16 August 2024)]. All-atom MD simulations were performed using GROMACS (*67*, *68*). The AMBERGS force field was used in simulations with SPC/E water model. The initial structures were placed in cubic boxes of size 84 Å and neutralized a strength of 150 mM counter Na^+^ and Cl^−^ ions. After ions neutralization, each system was subjected to energy minimization using the steepest descent algorithm until the maximum force achieving 1000 kJ mol^−1^ nm^−1^. Subsequently, the leap-frog integrator was used to maintain the temperature at 303.15 K in constant number of particles, volume, and temperature (NVT) equilibration, and maintain the pressure at 1 bar in constant number of particles, pressure, and temperature (NPT) equilibration. The electrostatic interactions were calculated using the Particle-Mesh Ewald algorithm, and cutoff distances for the calculation were set as 2.0 nm. Last, an MD run of 10 ns was executed for each system. Periodic boundary conditions were applied to all simulations and trajectory data were saved at time intervals of 10 ps.

### ICP-MS for intracellular metal levels measurement

Strains were harvested at OD_600_ = 0.6 and inactivation by autoclave records the weight after 60°C drying and filtration. After digesting by nitric acid in polytetrafluoroethylene tube using T0uchwin2.0, dilution by ultrapure water in polyethylene terephthalate plastic bottle was done. We detected metal levels through ICP-MS (PE NexION 300D) (*69*).

### Ethics statement

All applicable institutional and/or national guidelines for the care and use of animals were followed and all animal experiments were approved by the Ethical Committee for Animal Experiments of Xi’an Jiaotong University (no. XJTU-2020-35).
