# Supplementary material for: Molybdate uptake interplay with ROS tolerance modulates bacterial pathogenesis
Source: Sci Adv. 2025 Jan 15;11(3):eadq9686. doi: 10.1126/sciadv.adq9686 (PMC11734730; doi:10.1126/sciadv.adq9686)
Supplement: Supplementary file 1 — Figs. S1 to S5 Tables S1 to S3 [file sciadv.adq9686_sm.pdf]

Science Advances
AAAS

Supplementary Materials for

Molybdate uptake interplay with ROS tolerance modulates bacterial pathogenesis

Min Jiao *et al.*

Corresponding author: Yurong Wen, yurong.wen@xjtu.edu.cn

Sci. Adv. **11**, eadq9686 (2025)
DOI: 10.1126/sciadv.adq9686

This PDF file includes:

Figs. S1 to S5
Tables S1 to S3

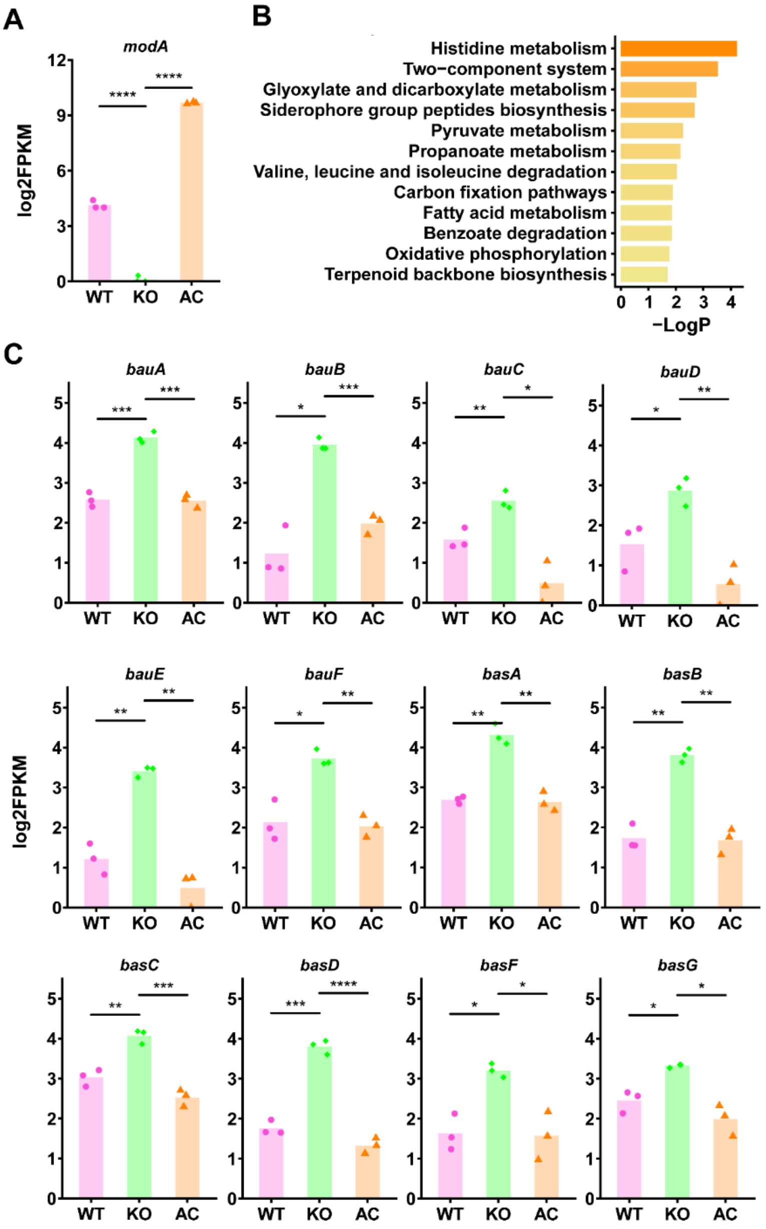

Supp. Fig. S1. ModA knock-out in *A. baumannii* ATCC 19606 disturbs metabolism.

(A) The expression pattern of *modA* in WT, KO and AC strains, no expression was detected in KO, while higher expression was observed in AC than WT.

(B) Rich distribution of DEGs in KEGG pathway demonstrated the diversity of metabolism affected by *modA* knock-out. Y axis represents different pathways, X axis represents P value.

(C) The same expression pattern of *bau* and *bas* in Fe uptake system in WT, KO and AC strains, all genes showed significantly up-regulated with *modA* deficiency.

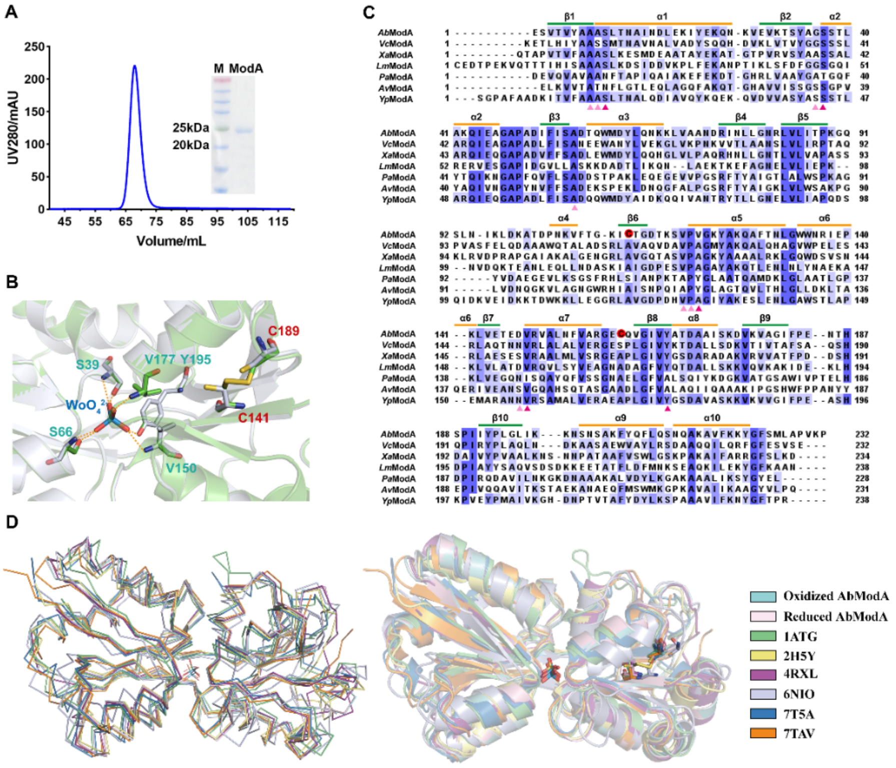

Supp. Fig. S2. ModA in *A. baumannii* possesses an unconventional disulfide bond. (A) ModA was purified through size exclusion chromatography Superdex 75pg, indicates monomeric elution. (B) Superposition of metal-binding pocket of tungstate -bound ModA in oxidized and reduced state is shown in ribbon representation. Residues interacting with the ligands are shown in stick representation and bonds between the structure and molybdate are shown by dashed lines. The hydro sulphonyl and disulfide bond from Cys141 and Cys189 are shown in yellow. (C) Sequences alignment of *Ab*ModA with other top 6 high similarity ModA from Dali search. The secondary structure is displayed above, with orange and green line represented for α helix and β strand. Cys is marked by red circle, directly and indirective interacting residues with molybdate are labeled by pink triangle and orange triangle, respectively. (D) The relatively conserved structure alignment of *Ab*ModA with other top 6 high similarity ModA from Dali search. The structures are displayed in stick form (left) and cartoon form (right), respectively, with the unique Cys 141 and Cys189 are labeled and the ligands are all shown in stick form.

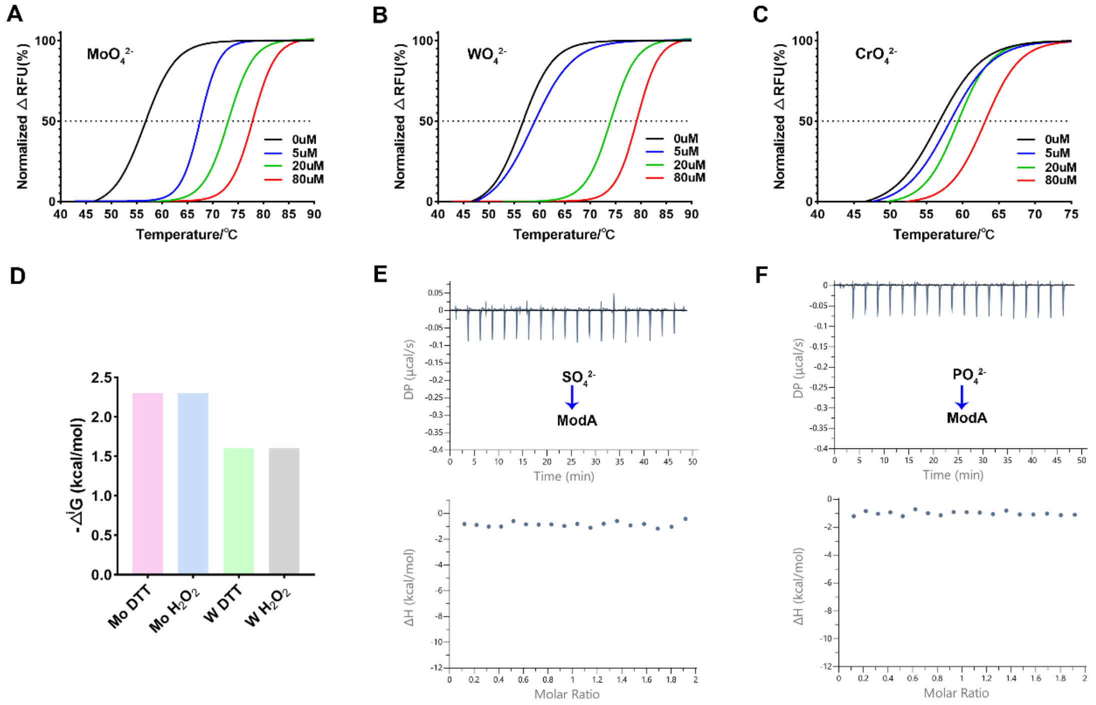

Supp. Fig. S3. ModA in *A. baumannii* ATCC 19606 interacts with Group VI oxyanions

(A-C) Heat shift assay of ModA to MoO_4^{2-}, WO_4^{2-} and CrO_4^{2-}. With the increase of the ligand's concentration from 0 μM, 5 μM, 20 μM to 80 μM, *Tm* changes were detected, with 56.58 °C, 67.51 °C, 73.09 °C and 77.91 °C for MoO_4^{2-}, 56.58 °C, 58.70 °C, 74.06 °C and 79.22 °C for WO_4^{2-} and 56.58 °C, 58.09 °C, 59.30 °C and 63.06 °C for CrO_4^{2-}, respectively.

(D) Bar graph of energy of ModA bound with molybdate and tungstate in oxidized and reduced state, showed higher energies of ModA coupled with molybdate than tungstate.

(E-F) Isothermal titration calorimetry (ITC) experiments of ModA to SO_4^{2-} and PO_4^{2-} demonstrated no interaction.

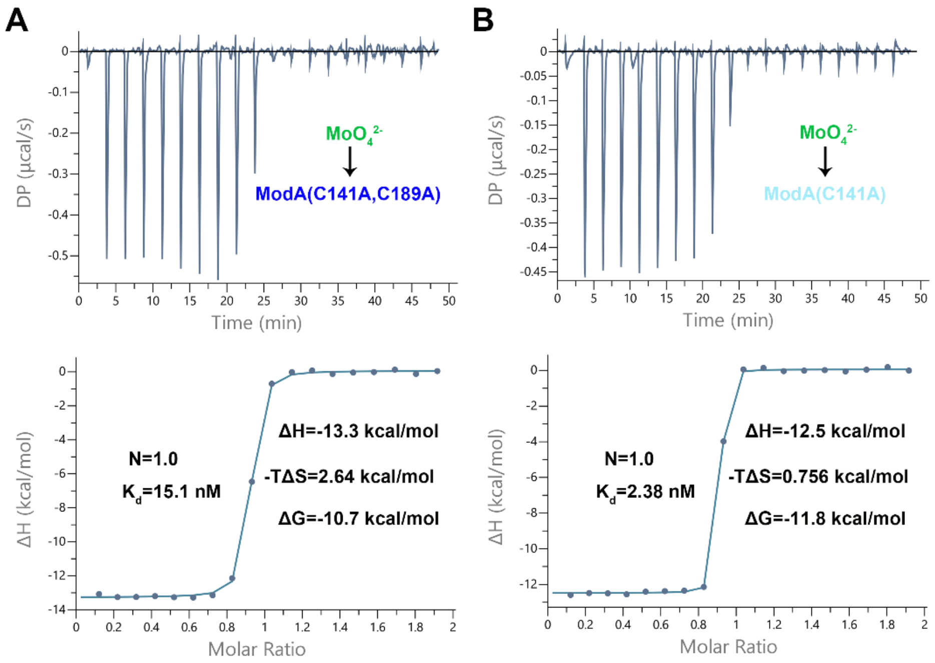

Supp. Fig. S4. Cystein141 and Cystein189 mutant increased the affinity of ModA to molybdate compared to ModA in oxidative (containing -S-S-) state.

Isothermal titration calorimetry (ITC) curves of ModA with both Cystein141 and Cystein189 mutant to alanine (A) and single Cystein141 mutant to alanine (B) against molybdate. The typical data was presented with three repeats performed.

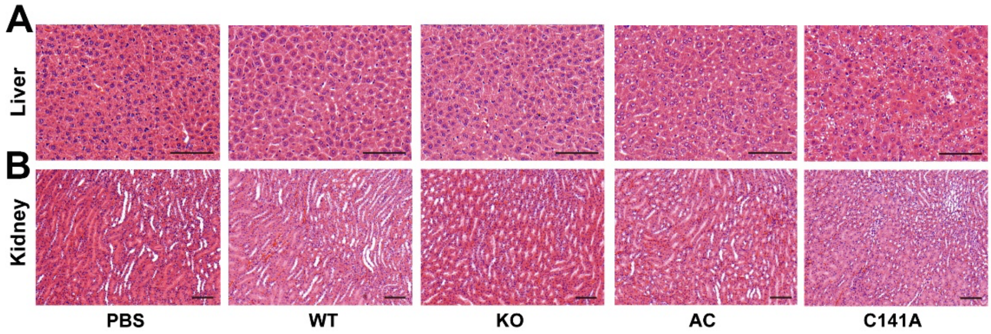

Supp. Fig. S5. *modA* knock-out in *A. baumannii* ATCC 19606 leads to no obvious damage to kidney and liver in mice infection model.

Histopathological analysis of the liver (A) and kidney (B) tissues showed no visible damage from WT, KO, AC, C141A and PBS infected mice for 1 day. All images are displayed with *Bars*= 100μm.

Supp. Table S1. *modABC* operon in *A. baumannii* ATCC 19606.

Gene ID	Gene Name	Size	Start	End	Strand
GO593_RS16370	RS16370	351	3418201	3418551	+
GO593_RS16375	*modA*	786	3418742	3419527	+
GO593_RS16380	*modB*	693	3419524	3420216	+
GO593_RS16385	RS16385	621	3420221	3420841	+

Supp. Table S2. X-ray data collection and refinement statistics.

Crystal	ModA MoO_4 DTT	ModA MoO_4 H_2O_2	ModA WO_4 DTT	ModA WO_4 H_2O_2
**Data collection**				
Spacegroup	C2	P1	C2	C2
a, b, c (Å)	85.6, 39.9, 79.3	47.3, 73.8, 79.1	85.6, 40.0, 79.3	86.1, 39.7, 79.4
α, β, γ (°)	90, 109.4, 90	97.7, 107.4, 100.6	90, 109.5, 90	90, 109.3, 90
Resolution (Å)	33.56-1.39 (1.44-1.39)	28-1.44 (1.49-1.44)	31.39-1.35 (1.40-1.35)	33.64-1.44 (1.49-1.44)
*R*merge	0.076 (0.738)	0.065 (0.935)	0.070 (0.278)	0.091 (0.675)
*R*meas	0.083 (0.841)	0.077 (1.138)	0.076 (0.310)	0.099 (0.746)
Multiplicity	6.4 (4.3)	3.5 (3.0)	6.4 (4.8)	6.5 (5.4)
CC(1/2)	0.996 (0.785)	0.998 (0.743)	0.998 (0.963)	0.998 (0.836)
CC*	0.999 (0.938)	1 (0.923)	0.999 (0.991)	0.999 (0.954)
I/σ(I)	11.7 (1.4)	7.5 (1.0)	12.7 (2.7)	9.6 (1.4)
Completeness (%)	97.98 (88.39)	92.08 (88.00)	92.33 (82.10)	98.52 (96.90)
Wilson B-factor ($Å^2$)	19.31	16.47	14.58	17.34
**Refinement**				
Total Reflections	319862 (19214)	581177 (47584)	328310(16352)	299495 (23859)
Unique Reflections	50299 (4493)	167285 (15781)	51740 (3436)	45779 (4387)
R_{work}/R_{free}	0.1620/0.1932	0.2561/0.2755	0.1667/0.1946	0.1768 /0.1860
Number of atoms:				
Protein	1750	7016	1744	1735
Ligands	5	20	5	5
Average B-factor ($Å^2$)	29.43	25.95	24.40	27.06
Protein ADP ($Å^2$)	27.42	24.97	22.78	25.33
Ligands ($Å^2$)	19.53	16.06	15.69	17.21
Ramachandran plot:				
Favored/Allowed(%)	98.67/1.33	98.23/1.77	98.11/1.78	07.77/2.33
Root-Mean-Square-Deviation:				
Bond lengths (Å)	0.006	0.007	0.008	0.010
Bond Angle (°)	0.87	1.05	0.96	1.15

Statistics for the highest resolution shell are shown in parentheses.

Supp. Table S3 Vectors, strains and primers used in this paper.

**1. Vectors**	
pET28a	pMMB67EH
**2. Strains**	
E. coli BL21 Star^{TM} (DE3), Thermofisher, Cat#: C601003	
Acinetobacter baumannii, ATCC, ATCC19606	
**3. Primers used in this paper**	
**AbModA pET28a construction**	
F-NcoI-AbModA-58℃	CATGCCATGGGCCATCATCATCATCATCACATGGAATCAGTTACTGTTTATGCAGCAG
R-EcoRI-AbModA-58℃	GGAATTCTCATGGCTTGACTGGAGCC
F-ModA-28a/pMMB-C141A-60.4℃	GCAAGATTgcTACAGGAGATACCAAAAGTGTGCC
R-ModA-28a/pMMB-C141A-61.4℃	CTCCTGTAgcAATCTTGCCTGTAAACACTTTATTAGGA
F-ModA-28a/pMMB-C189A-69.8℃	TGAAgcTCAGGTCGGTATTGTCTATGCAACCGA
R-ModA-28a/pMMB-C189A-60.4℃	ATACCGACCTGAgcTTCACCTCGCGCAACAAAG
**AbModA knock-out strains of Ab19606**	
F-AbModA-up-57.6℃	TTAGAGCAAAATATTGCATTTGG
R-Kan-AbModA-up-57.6℃	GAAGCAGCTCCAGCCTACACTATGTTTACCTTTTTGTCGTTTTTT
F-Kan-AbModA-down-56.4℃	GGACCATGGCTAATTCCCATTGTTTTCTTTAACCCCAGAAGA
R-AbModA-down-55.6℃	GGTGGCCCCAAACTCC
F-pET28a-AbModA-up-57.6℃	GCTTTGTTAGCAGCCGGATCTCAGTGTTAGAGCAAAATATTGCATTTGG
R-pET28a-AbModA-down-55.6℃	GTTTAACTTTAAGAAGGAGATATACCATGGGTGGCCCCAAACTCC
F-ID-AbModA-53.3℃	GCTATTTAAAACGTTAAATAAAAAC
R-ID-AbModA-53.2℃	CAATGAATGACTTACCCCAA
**AbModA pMMB67EH construction**	
F-pMMB-AbModA-Op-58℃	CGGATAACAATTTCACACAGGAAACATGGAATCAGTTACTGTTTATGCAGCAG
R-pMMB-AbModA-Op-58℃	CTTCTCTCATCCGCCAAAACAGTCATGGCTTGACTGGAGCC
